# In-vitro study of cytotoxic and apoptotic potential of *Thalassia hemprichii* (Ehren.) Asch. And *Enhalus acoroides* (L.f.) Royle against human breast cancer cell line (MCF-7) with correlation to their chemical profile

**DOI:** 10.1186/s12906-024-04512-3

**Published:** 2024-06-24

**Authors:** Shimaa I. A. Mohamed, Ghada H. Elsayed, Amgad El Shaffai, Shaymaa M.M. Yahya, Walaa S.A. Mettwally

**Affiliations:** 1 Chemistry of Natural and Microbial Products Department, Pharmaceutical and Drug Industries Research Institute, National Research Centre, ElBuhous St. 12622, Dokki, Dokki, Giza 12622 Egypt; 2 Nature Conservation Sector, Egyptian Environmental Affairs Agency (EEAA), Ministry of Environment, Cairo, Egypt; 3 Hormones Department, Medical Research and Clinical Studies Institute and Stem Cell Lab, Centre of Excellence for Advanced Sciences, National Research Centre, ElBuhous St. 12622, Dokki, Cairo, Giza Egypt

**Keywords:** Breast cancer, Seagrass, Apoptosis, Mitochondrial membrane potential, Cell cycle

## Abstract

**Background:**

Breast cancer is the most common type of cancer diagnosed in women. Finding novel therapeutic agents with significant cytotoxic action and minimal adverse impact on normal cells becomes crucial. Today, natural anticancer agents present an unconventional method of treating cancer, either as a curative or preventative agent, with considerable concern for marine organisms.

**Methods:**

The anticancer effect of the alcoholic extract of different Red Sea Seagrasses on MCF-7 human breast cancer cell line has been investigated. Seagrasses were collected from Wadi El Gamal, Red Sea and extracted. Qualitative HPLC analysis was performed on the extracts for the identification of their active biomarkers. This study was aimed to explore the cytotoxic impact of *Thalassia hemprichii* (Ehren.) and *Enhalus acoroides* (L.f.) Royle on MCF-7 and their mode of action. Their anti-proliferative effects on cancer cells were performed using Neutral red assay. On the other hand, their apoptotic effect and their capacity to induce cell cycle arrest were investigated by flow cytometry assay. The effect of Seagrasses on the mitochondrial membrane potential (ΔψM) was studied by using JC-1 mitochondrial membrane potential assay kit in Seagrasses treated cancer cells to Δψ Caspases 3/7activity was examined using the colorimetric method. Gene expression analysis and quantitative real time RT-PCR for the sea grasses on MCF-7 was performed. Immune-blotting technique for Bcl-2 and p53 was investigated.

**Results:**

HPLC analysis demonstrated that the extracts contained mainly flavonoids and polyphenols such as Caffeic acid, Chlorogenic acids, catechin and kaempferol that might be responsible for these anticancer effects. Seagrasses alcoholic crude extract markedly suppressed the growth and expansion of MCF-7 cells concentration-dependently with no toxicity against normal human skin fibroblast HSF. *Thalassia hemprichii* and *Enhalus acoroides* trigger mode of cell death primarily via apoptosis as confirmed by the flow cytometry. Additionally, they have ability to induce G0/S cell cycle arrest in MCF-7. The data showed the depletion in mitochondrial membrane potential (ΔψM) in the treated cells dose-dependently Caspases 3/7activities markedly increased following 24 h treatment. Finally, Gene expression analysis showed a marked reduction in Bcl-2, Survivin and CDC2 gene expression levels and a significant increase in the expression of p53 and CC2D1A as compared to control cells.

**Conclusion:**

In summary, the Methanolic extract of seagrass, *Thalassia hemperchii* and *Enhalus ocoroides* are able to induce concentration-dependent cytotoxic effects in human MCF-7 cells through intrinsic pathway of apoptosis in MCF-7 cells. This study reveals the beneficial importance of sea grasses as a source of anticancer agents. Further in vivo study is recommended for the active isolated biomolecules.

**Supplementary Information:**

The online version contains supplementary material available at 10.1186/s12906-024-04512-3.

## Introduction

Cancer is a fatal condition that arises from unregulated cell growth and expansion with a high potential to spread to other body organs [1]. Breast cancer is thought to be the main reason for cancer-associated death. Chemotherapy, radiation, and surgery are frequently used in the treatment of cancer [2]. The risk of cancer recurrence is still extremely significant, despite the high cure rate associated with surgical treatment [3]. In addition to having undesirable, severe side effects like hair loss, nausea, and vomiting, it has been reported that the majority of chemotherapeutic medicines cause toxicity to normal cells and can even lead to the development of resistance to the treatment itself [4]. Consequently, it becomes an imperative need to find new therapeutic agents with potent cytotoxic effect and no negative influence on normal cells. Nowadays, the natural anticancer agent represents a novel approach to cancer therapy either as a therapeutic or protective factor with a great concern for marine organisms [5]. Numerous chemically distinct substances originating from the sea have been studied and are being developed as novel nutraceuticals. One such example is Plitidepsin, which has been shown to be useful in treating a variety of malignancies, including melanoma, bladder, and small and non-small cell lung cancers [6, 7].

According to Kennedy and Björk [8], seagrass is the smallest taxonomic category of marine angiosperms, consisting of just around 60 species divided among four families and found worldwide, with the exception of the Polar Regions. The most fruitful meadow, which contains roughly 24 species, is made up of tropical seagrass [9]. They were historically ingested as supplements by the coastal people and utilized in the treatment of different remedies [10].

Marine seagrasses are a rich source for secondary metabolites, mainly, phenolic compounds [11]. *Thalassia hemperchii* is a widespread species of seagrass in the genus Thalassia that is indigenous to the shore of the Red Sea [12].

Yondelis® is the first marine-derived anticancer drug that received the European approval after clinical studies for the treatment of soft tissue sarcoma and ovarian carcinoma in 2007 and in 2009, respectively [13]. Phenolic compounds are one of the secondary metabolites of plants that play a role in their defense mechanism against different environmental conditions [14]. Marine organisms are a rich source of different classes of phenolic compounds [15]. Seagrasses are poorly studied widely spread marine plants that have been reported to have their capacity to induce cytotoxicity in various cancer cells via apoptosis as well as oxidative stress-dependent cell death [16, 17]. Based on this previous background, we decided to obtain and characterize crude MeOH extract from two different Seagrass to study their cytotoxic potential in breast cancer cells and their effect on mitochondrial membrane potential and caspases enzyme activities.

## Materials and methods

### Collection of seagrass material

Seagrass samples were gathered from the Red Sea, Egypt’s Wadi El Gemal National Park. Dr. Amgad ElShaffai (The Egyptian Environmental Affairs Agency (EEAA) carefully collected and identified the samples. *Enhalus acoroides* (Linn.F.) Royle was collected from Abu Ghoson Mangrove-North, Wadi El Gemal National Park, Marsa Alam, Egypt in February 2019. It is encompassing a segment of the Red Sea coastal plain (about 70 km of coastline, including the ecotourism development areas) and mountains extending roughly between 24°22’ N in the north and 24°05’ N in the south. The voucher specimens of the plant have been deposited in the Wadi El Gemal National Park herbarium under specimen number #WGAE0013. *Thalassia hemprichii* (Ehren.) Asch. was gathered from Sharm El Fakeri, Wadi El Gemal National Park, Marsa Alam, Egypt (Latitude 24°45 17.84” and Longitude 35°03 58.36”) in April 2018. Voucher specimen was deposited in the Wadi El Gemal National Park with specimen number # WGAE0012. Collection of living plants material from the wild was complied with Seagrass Net protocol and as per standards for seagrass collection, identification and sample design. Samples were thoroughly washed with sea water and epiphytes were carefully removed. Clean fresh samples were kept in freezer for further analysis.

### Method of extraction

Fresh *Enhalus acoroides* (Linn.F.) Royle and *Thalassia hemprichii* (Ehren.) Asch. were extracted as previously mentioned by [17]. Briefly, seagrass was extracted by methanol using electric blender, then the mixture was subjected for sonication for 20 min at room temp. Then, the mixture of each seagrass was filtered, and the solvent was evaporated under reduced pressure at 45 °C. The crude methanol extracts of *Thalassia hemprichii*, *Enhalus acoroides* were stored for further analysis.

### Qualitative HPLC analysis of phenolic

Phenolic acids and flavonoids were qualitatively analyzed using HPLC analysis. 10 µl of the crude MeOH extract (50 mg/ml, MeOH) was injected into HPLC (waters 2690 Alliance) with a photodiode array detector. The Column, C18 Intertsil ODS 3 (4.6 × 250 mm, 5 μm), was gradient eluted with a buffer of 0.1% phosphoric acid in water and methanol at a flow rate of 1 ml/min, ambient temperature and wave length 280 nm. Ten µl of each of the standard samples were injected into the HPLC under the same conditions after being dissolved in methanol.

### Fourier transform infrared (FT-IR) analysis

Potassium bromide (KBr) was mixed with a few mg of crude extract before being compressed into a disc with a 10 mm diameter. The Jasco FT/IR-6100 type A was used for the FT-IR analysis, with wave numbers ranging from 400 cm^− 1^ to 4000 cm^− 1^.

### Biological activities

#### Cell propagation and maintenance

The appropriate conditions were used to culture MCF-7 (cancer cell line) and HSF (normal human skin fibroblasts) cell lines obtained from the ATCC (American Type Culture Collection). The cells were propagated in DMEM (Lonza, Beligium) supplemented with 10% fetal bovine serum (FBS), 100 U/ml penicillin, and 100 g/ml streptomycin sulphate at 37 °C in a humid incubator with 5% CO_2_. The cells were collected after being trypsinized with 0.025% trypsin and 0.02% EDTA, followed by two washes in DPBS. Cells were divided for continued cultivation when the cell density reached about 80%. When the cells were in the logarithmic growth phase, the experiments were started.

### Anti- proliferation assay

Neutral red uptake assay was used to measure the cell viability [18]. The amount of viable cells in the culture can be quantitatively estimated using the neutral red uptake assay. It is predicated on how well-functioning cells can bind and integrate the supravital dye neutral red in lysosomes. At a cell density of 10^4^ cells/well in a 96-well plate, MCF-7 and HSF (normal cell line) were treated for 48 h with various concentrations of the crude extracts. 20 mg of both Methanolic extracts was dissolved in DMSO (Dimethylsulfoxide) and a serial dilution in culture media was prepared of different concentrations (0, 25, 50, 100, 200, 400, 800, and 1000 µg/ml). Similar to how the treated cells were cultured, a neutral red working solution (0.4 µg/ml) (Sigma-Aldrich) was incubated at 37 °C overnight. Culture media were removed from the cultured cells’ wells and neutral red medium (100 µl) was added to each well and incubated for 2 h to allow the vital dye to be incorporated into the viable cells. The Dulbecco’s PBS buffer (150 µl) was used to rinse the neutral red medium after removing them. By adding extraction buffer (150 µl, 1% acetic acid, 50% ethanol (96%) and 49% deionized water) and shaking the cells for at least 10 min on a micrometer plate shaker, dye was removed from the cells. In a micro-titer plate reader spectrophotometer (Sorin, Bio-medica S.p.A., Milan, Italy), the intensity of the extract’s neutral red color was measured at 530 and 645 nm as the excitation and emission wavelengths. Using the relationship between the neutral red and the used log concentrations, the IC_50_ of the tested crude extracts was determined. For the untreated cells (negative control), medium was added instead of the test crude extract. A positive control Doxorubicin (Dox, Mr = 543.5) was used (at concentrations 0.37, 0.75, 1.5, 3 and 6 µg/ml) as a cytotoxic agent giving 100% inhibition. Dimethyl sulfoxide (DMSO), the vehicle used for dissolving the tested crude extracts, was used and its final concentration on the cells was less than 0.2%. All experiments were done in triplicates and repeated 3 times under the same conditions. The data was statistically analyzed by graph prism V5 and presented as mean values with their standard error means (mean SEM).

cell viability % was calculated as follows:

$$\eqalign{& {\rm{Cell}}\;{\rm{viability}}\;\left( \% \right)\;{\rm{ = }}\;{\rm{OD}}\;{\rm{Treatment}}\;{\rm{ - }} \cr & {\rm{OD}}\;{\rm{blank}}\;{\rm{/}}\;{\rm{OD}}\;{\rm{control}}\;{\rm{ - OD}}\;{\rm{blank}}\;{\rm{*100}} \cr}$$

### Determination of cell death- annexin V / propidium iodide double staining apoptosis assay

The capacity of the tested extracts to induce necrosis and apoptosis was evaluated by using Annexin V-FITC apoptosis detection kit and two fluorescent channels flowcytometry (Abcam Inc., Cambridge Science Park, Cambridge, UK). MCF-7 cells were treated using three different concentrations 101.19 (IC_50_), 200 and 400 µg/ml for *Thalassia hemperchii* and 604.2 (IC_50_), 800 and 1000 µg/ml for ***Enhalus acoroides*** as well as Doxorubicin (1.2 µg/ml) as a positive control for 48 h. Following that, cells (1 × 10^5^ cells) were collected by trypsinization and washed twice with ice-cold PBS (pH 7.4). Cells were then incubated for 30 min at room temperature in the dark with 0.5 ml of Annexin V-FITC/PI solution, as directed by the manufacturer [19]. Cells were injected into the ACEA NovocyteTM flow-cytometer (ACEA Biosciences Inc., San Diego, CA, USA**)** after staining, and FITC and PI fluorescence signals were analyzed using FL1 and FL2 signal detectors, respectively (λex/em 488/530 nm for FITC and λex/em 535/617 nm for PI). For each test sample, 12,000 events were recorded, and the number of positive FITC and/or PI cells was determined using the ACEA Novo-Express TM software (ACEA Biosciences Inc., San Diego, CA, USA) and quadrant analysis.

### Assessment of cell cycle arrest

MCF-7 cells (5 × 10^5^ cells) were treated for 48 h with doxorubicin (1.2 µg/ml) (as a positive control) and extracts at IC_50_ concentrations (101.19 µg/ml for TH and 604.2 µg/ml for EA) for the measurement of cell cycle on a six-well plate at 37 °C and 5% CO_2_. Cells were collected and washed after trypsinization, centrifuged at 2000 ×g for 10 min., and the pellet was then re-suspended in 0.5 ml PBS. After adding 1.2 ml of 70% cold ethanol for two hours, fixation was finished. The fixed cells were then centrifuged at 2000x g for 10 min after being washed with PBS. 8 µl of DNAase free RNAse (10 mg/ml) was added and incubated for 1 h after cells had been suspended in 0.3 mL of PBS. Cells were incubated at 4 °C for 30 min after adding 15 µl of Propidium iodide (0.5 mg/ml). Using a flow cytometer FACS calibur (Beckman Coulter, Fullerton, CA, USA) with an excitation wavelength of 488 nm and an emission wavelength of 670 nm, the cells were examined for cell cycle [20]. ACEA Novo-ExpressTM software (ACEA Biosciences Inc., San Diego, CA, USA) was used to analyze cell cycle distribution and determine DNA content.

### Assessment of mitochondrial membrane potential

The mitochondrial membrane potential (ΔψM) in Seagrasses-treated MCF-7 cells was measures using the JC-1 mitochondrial membrane potential assay kit (ab113850, Abcam, Cambridge, United Kingdom) [21]. Tetra-ethylbenzimidazolyl carbocyanine iodide, a cationic dye that accumulates in polarized mitochondria attracted by a high ΔψM, generates red luminous aggregates at high concentrations. JC-1 ceases accumulation inside mitochondria upon ΔψM dissipation, and the low concentration monomers of JC-1 molecules show green fluorescence. We carried out the test in accordance with the manufacturer’s instructions. In a 96-well plate with a black wall, 15,000 MCF-7 cells were sown within each well before 100 µl of the treatment solution was applied. Both extracts were used at various concentrations (0, 100, 200, 400, 800 and 1000 µg/ml) for 48 h. Then, we added 100 µl of 2X JC-1 (40 µM) solution to each well 30 min before the end of the treatment, and we incubated the samples for 30 min at 37 °C in a light-protected environment. With 1X dilution buffer that had been pre-warmed (100 µl/well), the cells were washed twice. The final wash was left on the cells, and the microplate reader was used to detect the fluorescence of the aggregates (excitation: 475 nm, emission: 590 nm) and monomers (excitation: 475 nm, emission: 530 nm). We calculated the fold-change in relation to the negative control (untreated cells) using the aggregate-to-monomer fluorescence ratio (JC-1 ratio), which declines during ΔψM depolarization. Both JC-1 staining and final determination were carried out in the presence of test treatment. Thus, we spiked both staining solution and dilution buffer with the corresponding concentration of extract. The cells were treated with ionophore uncoupled FCCP (carbonyl cyanide 4-(trifluoromethoxy) phenylhydrazone, 50 µM) as a positive control for 2 h which decreased the JC-1 ratio to ~ 38% of the control (DMSO 0.1%) levels.

### Assay of caspase-3/7 activity

According to the manufacturer’s instructions (R&D Systems), a colorimetric assay kit was used to measure the enzymatic activity of the caspase 3/7 triggered by sea grasses [22]. Briefly, Cells 1 × 10^5^/well were treated with doxorubicin (1.2 µg/ml) (as a positive control) and extracts at IC_50_ concentrations (101.19 µg/ml for TH and 604.2 µg/ml for EA) for 24 h. After the treated cells were lysed in a lysis buffer, the protein was incubated at 37 °C for 2 h with 50 µl of a reaction buffer and 5 µl of the colorimetric tetrapeptide Asp-Glu-Val-Asp (DEVD)-p-nitroaniline (pNA) for caspase-3/7. At a wavelength of 405 nm, the optical density of the reaction mixture was measured spectrophotometrically.

### Immunoblotting

In 6 well culture plate, 1 × 10^5^/well cells were treated with doxorubicin (1.2 µg/ml) (as a positive control) and extracts at IC_50_ concentrations (101.19 µg/ml for TH and 604.2 µg/ml for EA) for 24 h. RIPA buffer was used to obtain the cell lysates from MCF-7 cells that had been pre-treated for 48 h. Thermo-Fisher scientific’s Pierce BCA Protein assay kit was used to calculate the total protein concentration. Following 10% SDS-PAGE (sodium dodecyl sulphate polyacrylamide gel electrophoresis), samples of the same protein content (30 µg/20 µl) were blotted onto polyvinylidene difluoride membranes (PVDF) (Millipore, Bedford, MA, USA). The membrane was first blocked for 1 h at 25 °C with 5% non-fat milk in TBS-T buffer containing 0.1% Tween 20 before being incubated overnight at 4 °C with blocking solution containing primary antibodies for Bcl-2, p53 and GAPDH using rabbit polyclonal anti-Bcl-2 (# PA5-20068, Invitrogen, Thermo Fisher Scientific, USA), mouse monoclonal anti-p53 (# MA5-12557, Invitrogen, Thermo Fisher Scientific, USA) and rabbit monoclonal anti-GAPDH (#2118S, Cell Signaling Technology, USA) at 1:1000 dilutions, respectively. The membrane was then washed three times before being incubated for 1 h at 25 °C with constant agitation in the blocking solution containing anti-mouse and anti-rabbit secondary antibody conjugated to horseradish peroxidase (HRP) at 1:3000 dilutions (Cell Signaling Technology, USA). The positive results were acquired using an enhanced chemiluminescence (ECL) system (Amersham Pharmacia Biotech) following the washing phase. Using ChemiDoc imaging system of Bio-Rad Co, protein bands were detected. The expression level of protein was quantified using Bio-Rad Image Lab Software. The internal control used was the GAPDH expression.

### Gene expression analysis

RNA was extracted and isolated from cells treated with both extracts (101.19 µg/ml for TH and 604.2 µg/ml for EA) and Doxorubicin (1.2 µg/ml) using Qiazol buffer (Qiagen, USA), as directed by the manufacturer. RNA-easy Mini-Kit (Qiagen, USA) was then used to clean up the RNA. Using the easyscript first strand cDNA synthesis supermix (Transbiotech, China), RNA was reverse transcribed. The copy numbers of b-actin, Bcl-2, survivin, CDC2, p53, and CC2D1A were measured using Transbiotech’s perfect start green qpcr supermix (China). The housekeeping gene beta actin was used to normalize the copy numbers to 100,000 copies. Table 1 contains a list of primer sequences. The following were the RT and subsequent PCR cycling conditions: RT: 42 °C for 10 min, 85 °C for 5 s; qPCR: 94 °C for 30 s, 94 °C for 5 s, then 60 °C for 30 s.; there were 40 cycles total. Bio-Rad Real Time Thermal Mini-Opticon ^TM^ Thermal Cycler was used to quantify the gene expression.

**Table 1:** Primers used for gene expression analyses

Gene	Primer forward (5’-3’)	Primer reverse (5’-3’)	
**β-actin**	CCTTCCTGGGCATGGAGTCCT	GGAGCAATGATCTTGATCTTC	
**BCL-2**	CCTGGTGGACAACATCGCC	AATCAAACAGAGGCCG CATGC	
**Survivin**	AGGACGGCCCTTCTTGGAGG	CTTTTTATGTTCCTCTATGGGGTC	
**CDC2**	CAAATATAGTCAGTCTTCAGGAT G	CCTGTAGGATTTGGTATAAATAAC	
**p53**	AGA GTC TAT AGG CCC ACC CC	GCT CGA CGC TAG GAT CTG AC	
**CC2D1**	GTGGATGTCGCTGAATTGCC	GTGGATGTCGCTGAATTGCC	

### Statistical analysis

Data from three different trials are represented as mean values with their standard error means (mean SEM). All data were analyzed using Graph Prism v5. One-way analysis of variance (ANOVA) and post-test Dunnett’s multiple comparison tests were used to analyze the data. For all tests, statistical significance was defined as *P  < 0.05. All experiments were done in triplicates and repeated under same conditions.

## Results

### HPLC analysis

**Fig. 1:**
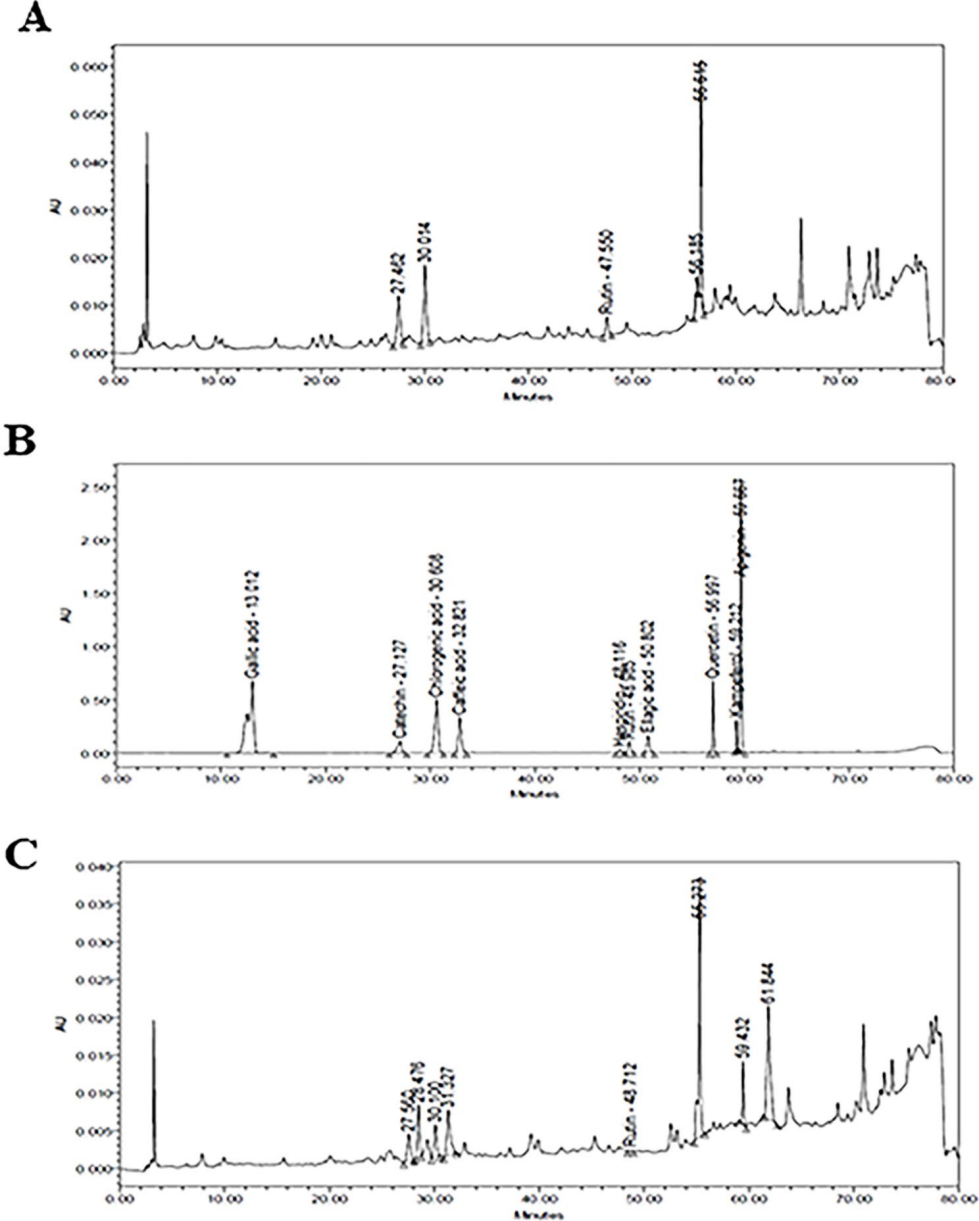
HPLC analyses of MeOH extract of (**A**) Enhalus acoroides (L.f.) Royle acoroides (Linnaeus f.) and (**B**) Thalassia hemprichii (Ehrenberg). a Representative HPLC chromatograms of (**C**) mixture of standards for identification of phenolic acids mainly (1) Chlorogenic acid (RT 30.608 min), (2) Caffeic acid (RT 32.821 min) and flavonoids mainly, (1) Catechin (RT 30.608), (2) Rutin at (RT 48.985 min), (3) Quercetin at (RT 56.997 min), (4) Kaempferol at (RT 59.212 min), (5) Apignin at (RT 59.667 min)

HPLC analysis (Table 2) reveals the abundance of Chlorogenic acid, Catechin, rutin and quercetin in both sea grass. On the other hand, Caffeic Acid, Kaempferol and Apigenin were only detected in *Thalassia hemprichii* (Ehern.) Asch. Seagrass were reported as a valuable source of phenolic compounds [23]. Chlorogenic acid (caffeoylquinic acid) is first to be detected in seagrass while other phenolic acids and flavonoids were previously isolated and/or detected. Caffeic acid was isolated from the alcoholic extract of *Thalassia hemprichii* (Ehren.) Asch [24, 25] and was detected only in the leaves of *Enhalus acoroides* (L. f.) Royle [15]. Rutin was previously detected using HPLC in leaves, roots and rhizome of *Enhalus acoroides* (L. f.) Royle. While Catechin was only in rhizome [17]. Quercetin, Kaempferol and Apigenin, were detected for the first time (Fig. 1). In our study apigenin wasn’t detected in *Enhalus acoroides* (L. f.) Royle that was in contrary to [26] Qi et al., 2008 findings. Further fractionation and purification is required to illustrate more phenolic compounds.

**Table 2:** Identification of the major compounds in *Enhalus acoroides* (L.f.) Royle and *Thalassia hemprichii* (Ehren.) Asch. by HPLC qualitative assay

Standard samples	Enhalus acoroides	Thalassia hemprichii
**Phenolic acids:**		
**Gallic Acid**	-	-
**Ellagic acid**	**-**	**-**
**Chlorogenic acid**	**+**	**+**
**Caffeic Acid**	**-**	**+**
**Flavonoids**:		
**Rutin**	+	+
**Catechin**	+	+
**Hesperidin**	-	-
**Quercetin**	+	+
**Kaempferol**	**-**	**+**
**Apigenin**	**-**	**+**

### FTIR analysis

**Fig. 2:**
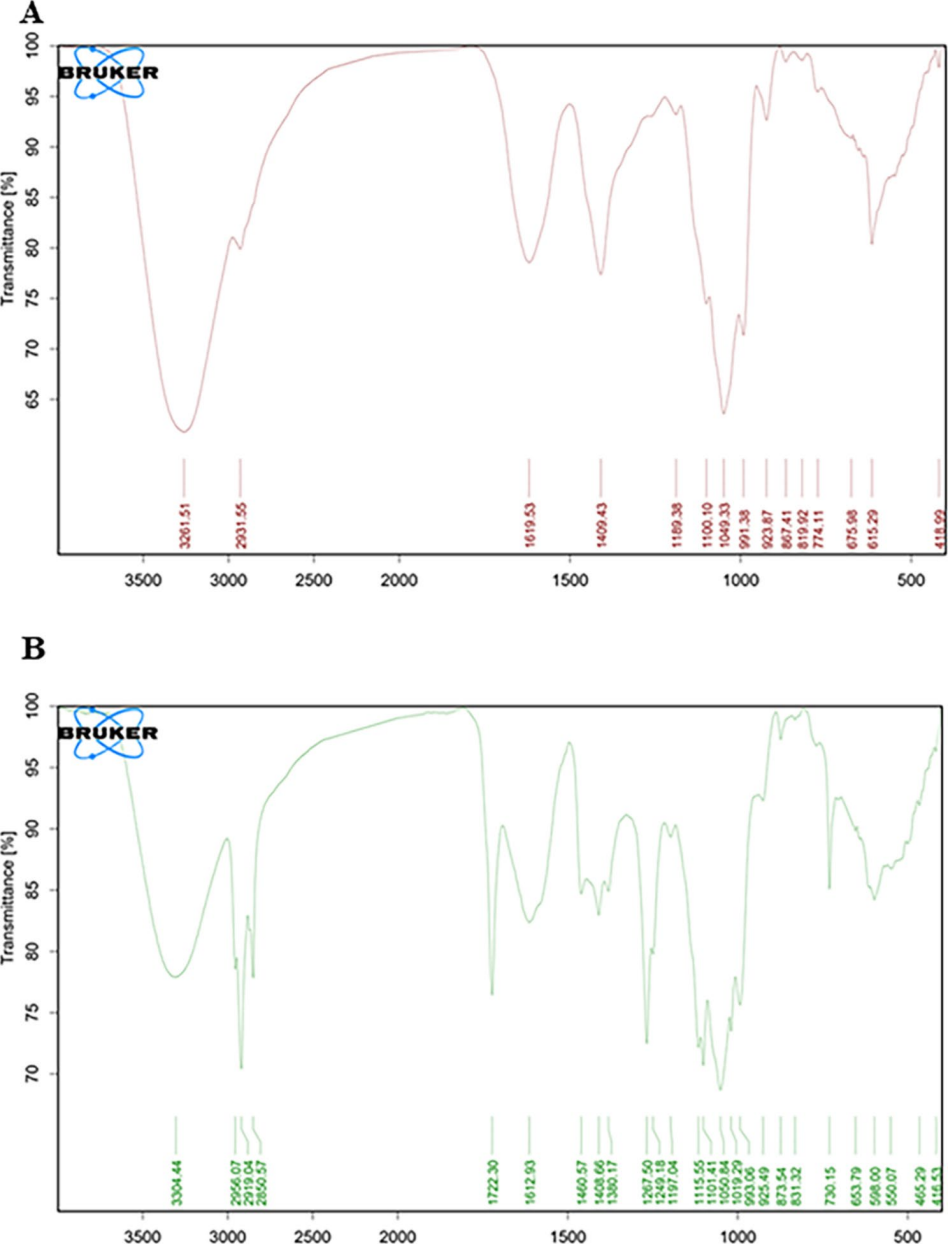
FTIR spectrum of (**A**) *Enhalus acoroides* (L.f.) Royle and (**B**) *Thalassia hemperchii*

FTIR analysis was performed to detect the functional groups. The spectra (Fig. 2A and B) were interpreted and presented in Table 3 *Thalassia hemprichii* (Ehren) Asch spectrum illustrates the presence of alkanes, cyclic alcohol, aromatic amine III, hydroxyl, amide, and ketone functional groups that were absent in *Enhalus acoroides* (L.f.) Royle. Those functional groups are attributed to the abundance of terpenoids and flavonoids (Philip et al., 2011). 2850 and 2919 cm^− 1^, symmetric and asymmetric stretching vibrations of methylene (-CH2), respectively, 1722, 1380 cm-1 C = O or C-H stretching, and 1460 cm^− 1^ asymmetric (δ) bending vibration of C-H and the stretching vibration of aromatics corresponding to CH3, CH2 of flavonoids and aromatic rings.

Both spectra show a broad, strong band at 3261 and 3304 cm^− 1^, revealing OH-stretching vibration corresponding to phenols or alcohol [27]; the latter may also be attributed to lipids [28]. Spectra also illustrate C-N stretching bands that reveal the presence of amines, amides, and proteins. C = C stretching vibration ring (1409.43 cm^− 1^) attributed to flavonoids and amino acids. *Enhalus acoroides* (L.f.) Royle spectrum, characterized by a band at 1189.38 cm^− 1^, reveals the presence of sulfated compounds. The FTIR confirms the HPLC finding that *Thalassia hemprichii* (Ehren.) Asch has higher phenolic compounds content than *Enhalus acoroides* (L.f.) Royle, which directly correlated to the difference in their anticancer activity.

**Table 3:** FT-IR spectral analysis of alcoholic extract of *Enhalus acoroides* (L.f.) Royle (EA) and *Thalassia hemprichii* (Ehren.) Asch (TH)

EA	TH		Functional group	Reference
**490–620**		C–Br, C-I	Halogenated derivative	[29]
**610–690**		-C-H wagging vibration	w, Vinyl hydrocarbon compounds	[30]
**-**	730.15	OH vibration	W, benzene derivative	[29]
**774.11**	-	-C-H bending vibration	Benzenoid	[30]
**819.92**	-	C-O-C, symmetric stretch	St.br, aromatic ethers	[29]
**-**	831.32	C-C skeletal vibration	w, Alkane	
**867.41**	-	R-NH2	w, primary amine	[31]
**923.87**	925.49	OH bending	w, s, carboxylic acid	[32]
**991.38**	993.06	C = C skeletal vibration	m, s,alkene	
**-**	1019.29	CH-O-H in cyclic alcohols	cyclic alcohols	[31]
**1049.43**	1050.84	CH2 and CH –O-H	St, br, Primary alcohol	[31]
**1100.10**	1101.41	C-O-H strectching vibration	St, s secondary and tertiary alcohols	[31]
**1189.38**	1197.04	C-N stretch	w, amine	
**-**	1267.50	C-N stretch ring	St, s, aromatic amine	[33]
**-**	1249.18	C-O-C	S,- alky and aryl ethers	[31]
**-**	1380.17	CH deformation	cellulose and hemicellulose	
**1409.43**	1408.66	C-H bending coupled with C-C stretching	m, s - Secondary alcohol-free	[30, 34]
**-**	1460.57	Asymmetric CH- bending	m, R-CH3, R- CH2	[29]
**1619.53**	1612.93	C = C conjugated with C = O or N-H bending	α, β-unsaturated ketone, cyclic alkane, amides	[27]
**-**	1722.30	C = O stretching	S, aliphatic ketone, carboxylic acid, α, β-unsaturated ester	
**-**	2850.57	Symmetric CH stretching	m, Alkane lipid	[35]
**-**	2919.04	Asymmetric CH2stretching	st, s, alkanes lipid	[35]
**2931.55**	-	OH stretch	w, br, alcohol	
**-**	2956.07	Asymmetry CH3 stretching	alkanes and lipid	[29, 35]
**-**	3304.44	Aromatic (-OH)	S, phenol	[36]

*m = medium, st = strong, br. = broad, w = weak, s = sharp

### Biological assays

#### In vitro evaluation of cytotoxic activity

In this experiment, we investigated the cytotoxicity of two different Seagrasses including *Enhalus acoroides* (Linnaeus f.) Royle and *Thalassia hemprichii*. Using the neutral red assay, which is based on the ability of viable cells to incorporate and bind the supravital dye neutral red in the lysosomes, the cytotoxic activity of the crude methanolic extracts was investigated individually as anti-cancer agents against human breast cancer (MCF-7) cells at different concentrations (0, 50, 100, 200, 400, 800, and 1000 µg/ml). The reference drug, doxorubicin, has IC_50_ values of 1.2 µg/ml against the tested cell line MCF-7. After being exposed to DMSO for 48 h, MCF-7 vitality was not significantly affected. The studied extracts markedly inhibited cell proliferation as compared to negative control cells.

**Fig. 3:**
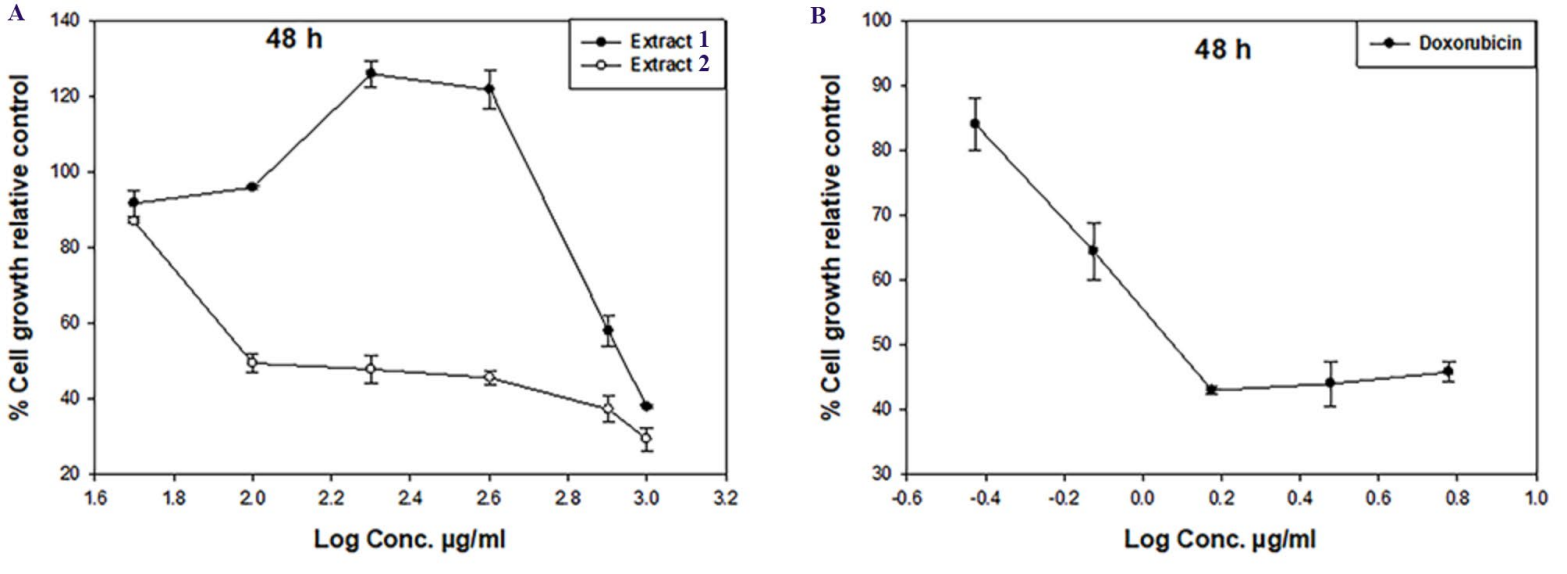
Cytotoxic effects of (**A**) extracts *Enhalus acoroides* (1), *Thalassia hemperchii* (2) and (**B**) Doxorubicin on MCF-7 cells at 48 h

Data in Table 4 and Fig. 3 (A, B) indicated that the cytotoxic activity of the tested crude extracts was against MCF-7 cancer cells. The data showed that the cell viability of the treated cell line decreases dose-dependently. At 48 h, *Thalassia hemperchii* (101.19 µg/ml) showed more inhibitory effect. On the other hand, *Enhalus acoroides* revealed inhibition on the growth of MCF-7 with IC_50_ values of 604.2 µg/ml. Both extracts are safe and don’t exhibit any cytotoxicity on normal human skin fibroblast (HSF).

**Table 4:** IC _50_ values of different Sea grasses *Enhalus acoroides* and *Thalassia hemperchii* on human breast cancer MCF-7

Compounds	IC_50_ (µg/ml)
**Doxorubicin**	1.2
***Enhalus acoroides *** **(1)**	604.2
***Thalassia hemperchii *** **(2)**	101.19

### Thalassia Hemperchii and Enhalus acoroides induce programmed cell death

**Fig. 4:**
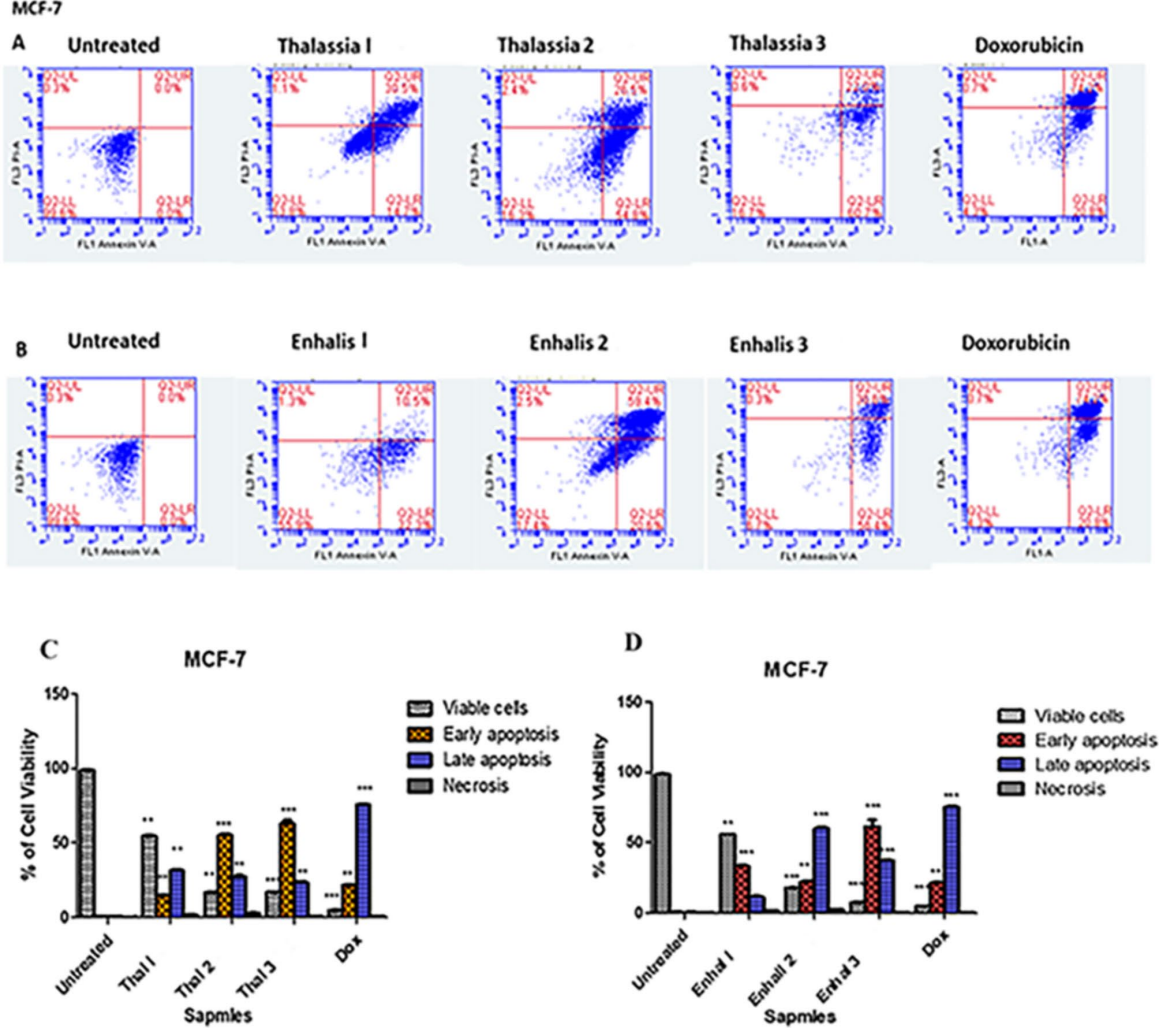
Representative FACS analyses of Annexin V and PI staining. MCF-7 cells were treated with (**A**) *Thalassia hemperchii and* (**B**) *Enhalus acoroides* for 48 h. Percentage of early and late apoptosis increased after treatment with 3 different concentrations (Thal 1 = 101.19, Thal 2 = 200 and Thal 3 = 400 µg/ml and Enhalus 1 = 604.2, Enhalus 2 = 800 and 1000 µg/ml) in compare with untreated cells. Bar graphs represent the percentage of viable, early apoptosis, late apoptosis and necrosis cells after treatment with (**C**) *Thalassia hemperchii and* (**D**) *Enhalus acoroides for 48 h.* Each histogram represents the mean ± SD from three independent experiments. * P < 0.05 and **P < 0.01 ***P  < 0.001 compared with the control, as determined by one-way ANOVA

To explore the capability of *Thalassia hemperchii* and *Enhalus acoroides* to induce cell death via apoptosis rather than necrosis, MCF-7 cells were pre-treated using three different concentrations 101.19 (IC_50_), 200 and 400 µg/ml for *Thalassia hemperchii* and 604.2 (IC_50_), 800 and 1000 µg/ml for *Enhalus acoroides* as well as Doxorubicin as a positive control. The percentage of early and late apoptosis was determined by Annexin V/PI dual staining kit following 48 h of treatment. In *Thalassia hemperchii* treated MCF-7 the percentage of early apoptosis increased from 14.7% at IC_50_ value up to 60.7% by treatment with 400 µg/ml (Fig. 4A & C). Furthermore, the percentage of late apoptosis increased significantly up to 22%. On the other hand, *Enhalus acoroides* induced apoptosis in MCF-7 treated cells with marked increasing in early and late apoptosis up to 56.4% and 59.4%, respectively (Fig. 4B & D). The data shows that both extracts are able to induce programmed cell death after treatment with IC_50_ concentration rather than necrosis.

### Effect of *Thalassia hemperchii* and *Enhalus acoroides* on cell cycle distribution

**Fig. 5:**
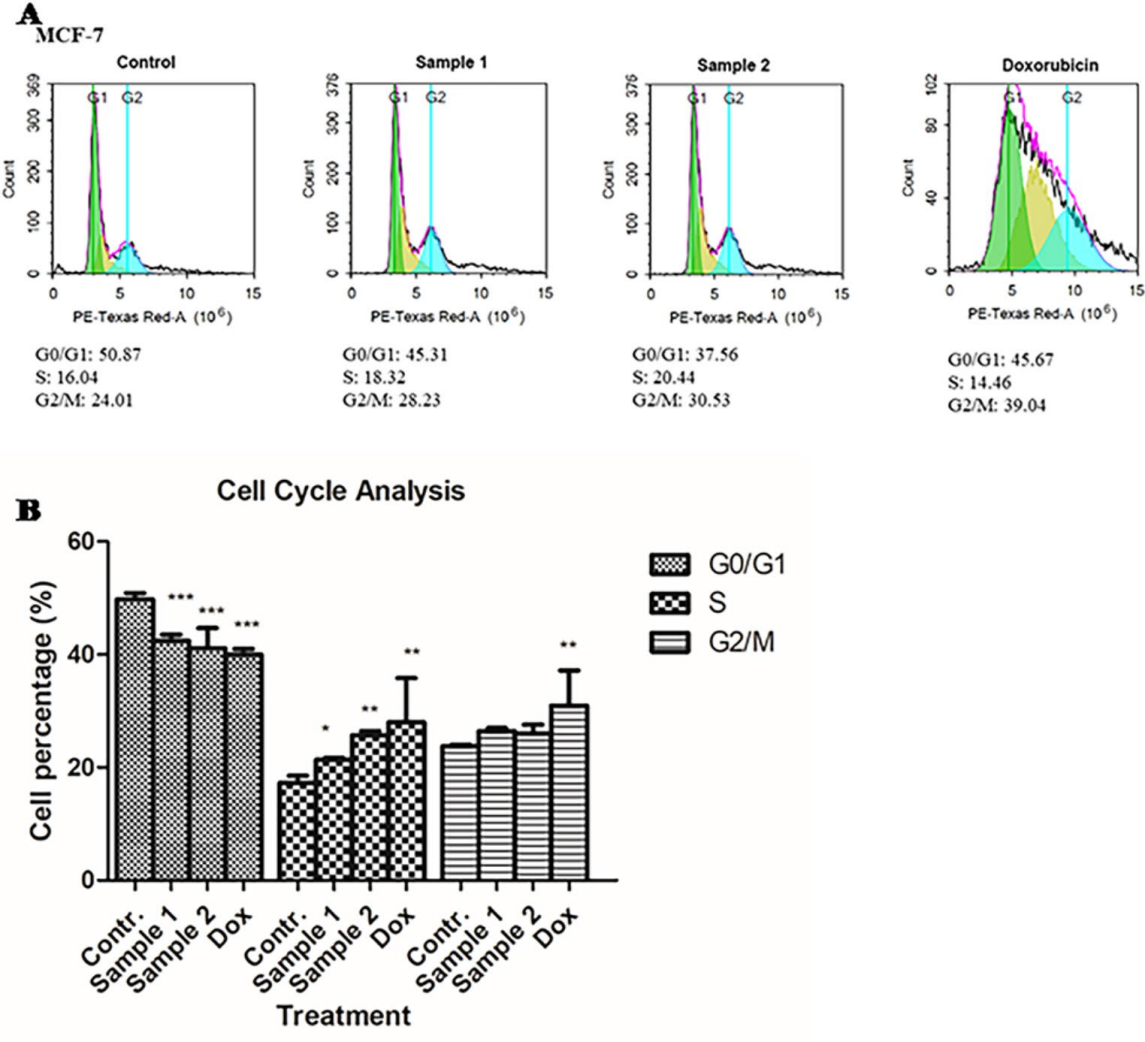
(**A**) Cell cycle analysis by flow cytometry. MCF-7 cell line was treated with *Enhalus acoroides (Sample 1), Thalassia hemperchii* (Sample 2) and Doxorubicin as a positive control. (**B**) Percentage of cell populations in MCF-7 treated cells with *Enhalus acoroides* (Sample 1), *Thalassia hemperchii* (Sample 2) and Doxorubicin as a positive control. Data are expressed as mean ± SD. (%). The data show a significant decrease *** *P* < 0.001, * * P  < 0.01 in the percentage of cells at G0/G1 phase in treated sample in compare with negative control (untreated cells)

The capacity of sea grasses extracts to inhibit cells proliferation was further evaluated by determination of cell cycle distribution. After 48 h of treatment using the methanol extract of *Thalassia hemperchii* and *Enhalus acoroides* (101.19 and 604.2 *µ*g/ml, respectively) and Doxorubicin (1.2 *µ*g/ml) or control cells with vehicle (< 0.5% DMSO), an increase in S phase concomitant with a significant decrease in G0/G1 phase (*** *P* < 0.001) was observed as compared with untreated cells (Fig. 5A& B). The G2/M phase did not significantly increase following extract treatment. The data suggests that G0/S phase cell cycle arrest is related to the growth suppression of MCF-7 cells by methanol extract. The expression levels of pro-apoptotic Bcl-2 and anti-apoptotic p53 were measured utilizing western blots and gene expression patterns to further analyze these findings [37].

### Mitochondrial membrane potential

**Fig. 6:**
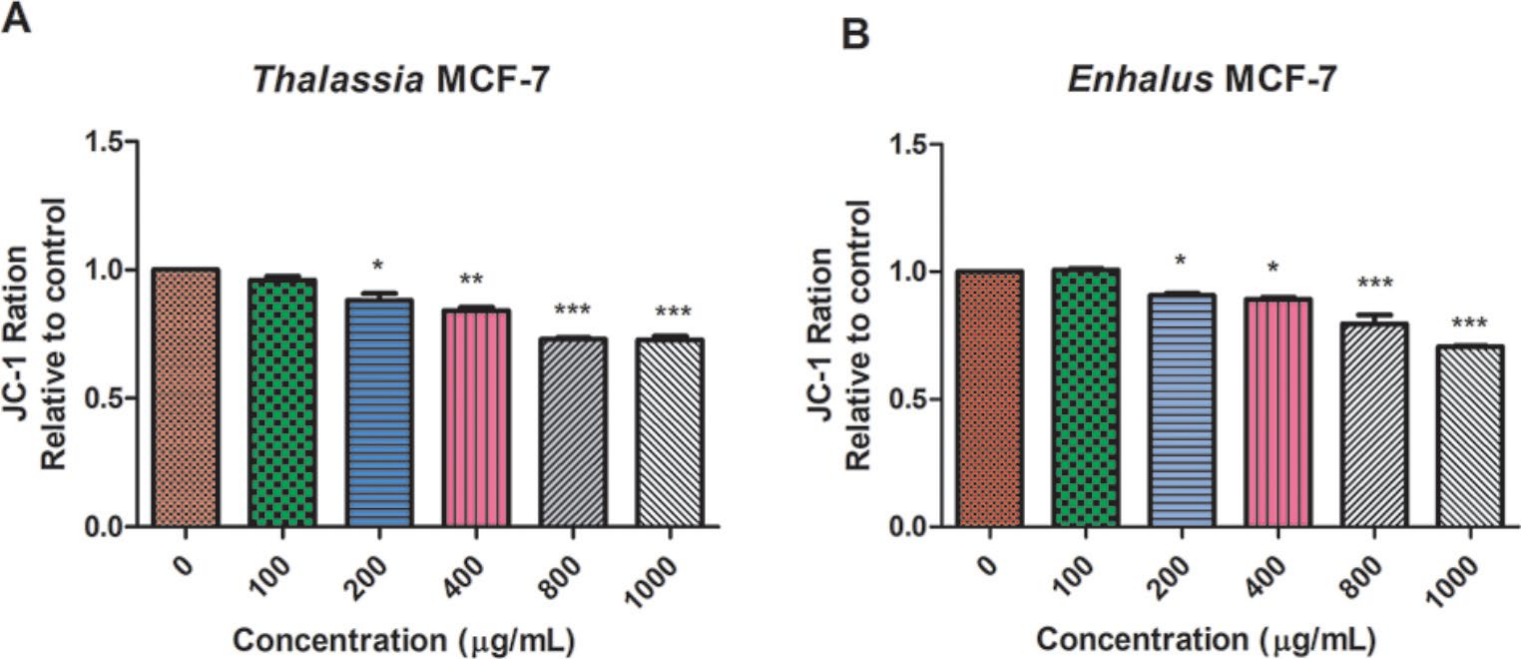
Effect of (**A**) *Thalassia hemperchii* (**B**) *Enhalus acoroides* on mitochondrial membrane potential of MCF-7. Mitochondrial membrane potential (Δψ_M_) was measured by JC-1 fluorescence [fluorescence of JC-1 monomers (em 535 nm)/aggregates (em 590 nm)] in MCF-7 cells treated with (100, 200, 400, 800 and 1000 µg/mL) for 48 h. *Thalassia hemperchii* and *Enhalus acoroides* showed significant decrease in MCF-7 cells. Data are expressed as mean ± SD. *** *P* < 0.001, * *P* < 0.05

*Thalassia hemperchii* and *Enhalus acoroides* were used at various concentrations (0, 100, 200, 400, 800 and 1000 µg/ml) to treat the MCF-7 cell line in order to measure the mitochondrial membrane potential. The result demonstrated that there was a drop in mitochondrial membrane potential (Δψ_M_) in the treated cells in dose-dependent manner. The results showed that *Thalassia hemperchii* was more significant in induction of mitochondrial membrane potential depletion in MCF-7 than *Enhalus acoroides.* Treatment of the MCF-7 cells with the *Thalassia hemperchii* and *Enhalus acoroides* for 48 h induced a reduction in mitochondrial membrane potential (depolarization), expressed as the reductions in JC-1 590/530 nm fluorescence ratios (in control versus in cells treated with the extract) (Fig. 6), which is a crucial indicator of mitochondrial-dependent apoptosis. We calculated the fold-change compared to the negative control (untreated). The ratio between aggregate and monomer fluorescence (JC-1 ratio) was determined, which declines with Δψ_M_ depolarization.

#### Induction of caspase 3/7 activity

**Fig. 7:**
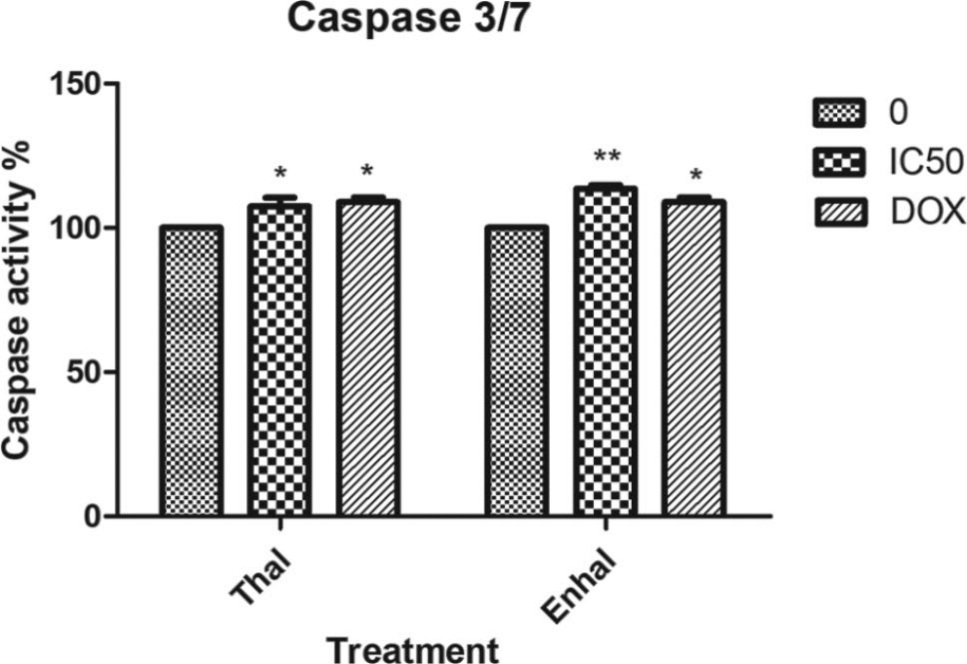
Activity of caspase-3/7 in MCF-7 cells after 24 h treatment with IC50 of samples *Thalassia hemperchii* and *Enhalus acoroides.* Each histogram represents the mean ± SD from three independent experiments. *P  < 0.05 and **P  < 0.01 compared with the control, as determined by one-way ANOVA.

Caspases are activated at the last stage of apoptosis [38], and understanding the active pathway is crucial for developing more effective treatment strategies [39]. In the current investigation, the activation of caspase-3/7 was examined on the MCF-7 cell line at concentrations of 101.19 (IC_50_) for *Thalassia hemperchii* and 604.2 (IC_50_) for *Enhalus acoroides* extracts after 24 h. At IC_50_ values, the MCF-7 cells’ caspase-3 activity was considerably higher than that of the control (untreated) cells (Fig. 7). The cleavage of various caspases results in the execution of apoptosis. We gain insight into cell death and other biological processes by better understanding the effects of caspase cleavage [40]. According to Lim et al. [41], the caspase activity in treated MCF-7 and disturbance in the mitochondrial membrane potential indicate that the extracts cause apoptosis via an intrinsic mechanism.

### Western-blot analysis for the expression of Bcl-2 and p53 in MCF-7

**Fig. 8:**
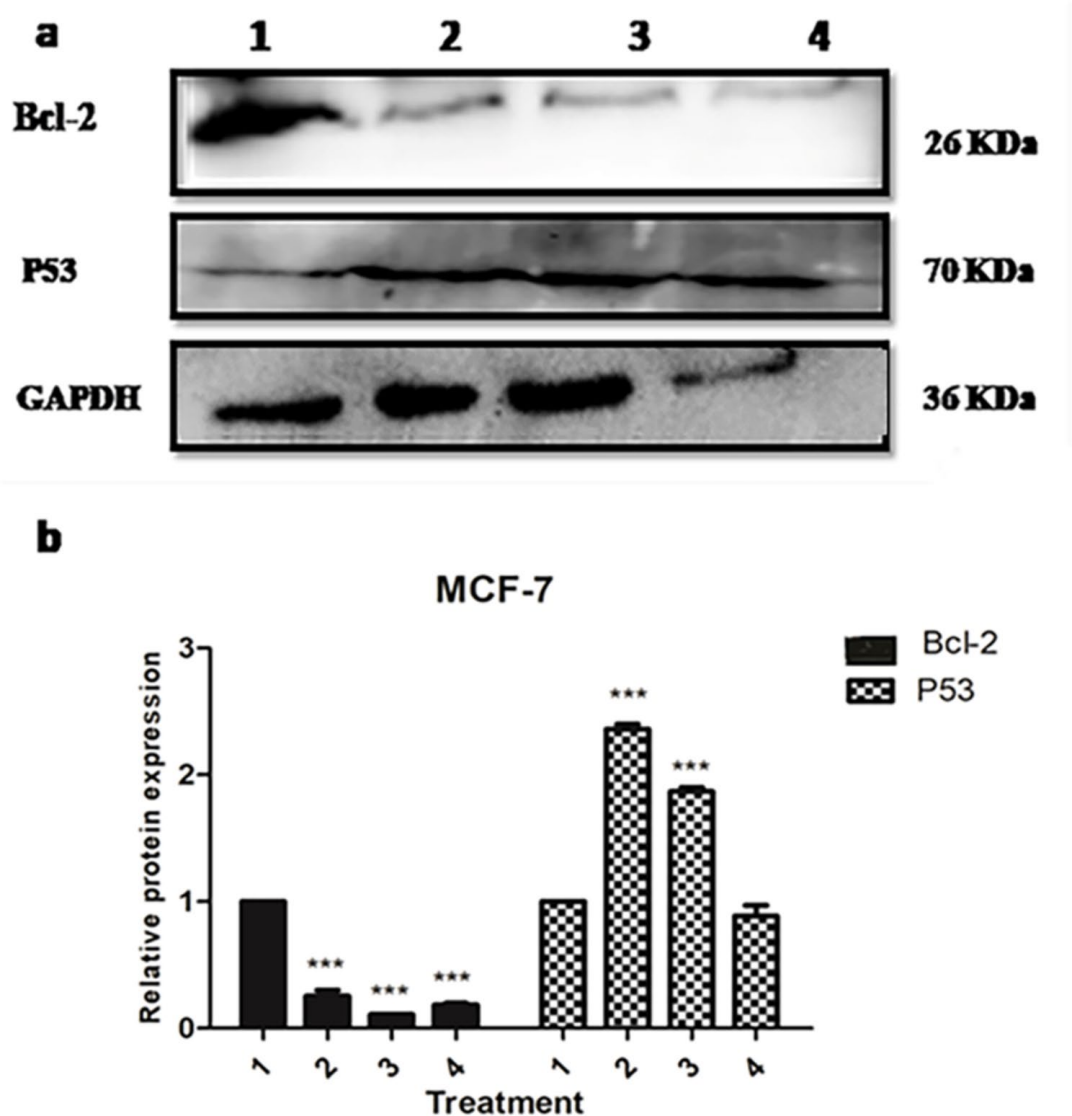
(**a**) Western blot analysis and (**b**) quantification of protein expression level of Bcl-2 and p53 in MCF-7 after treatment with 2 (*Thalassia hemperchii at* 101.19 µg/ml), 3 (*Enhalus ocoroides at* 604.2 µg/ml) and 4 (Doxorubicin at 1.2 µg/ml) for 48 h relative to GAPDH and compared with (1) untreated cells. All samples were derived from the same experiment and the blots were processed in parallel. Data are expressed as mean ± SD. ***P  < 0.001. The expression level of protein was quantified using Image Lab Software Bio-Rad

The expression levels of the apoptotic markers, p53 and Bcl‑2 protein, in MCF‑7 cells were evaluated using western blot technique. MCF-7 cells were treated with both sea grasses *Thalassia hemperchii* and *Enhalus acoroides* with IC_50_ values (101.19 and 604.2 µg/ml, respectively). The results indicated that the expression of Bcl‑2 was significantly decreased in the treated cells (*** *P* < 0.001) (Fig. 8a & b). On the other hand, extracts increased the expression levels of p53 compared with the untreated control cells (Fig. 8a & b). Doxorubicin was used as a positive control with IC_50_ value of 1.2 µg/ml. Natural products rich in kaempferol and caffeic acid induce cytotoxic effect against breast cancer MCF-7 via induction of apoptosis [42, 43].

#### Gene expression patterns

**Fig. 9:**
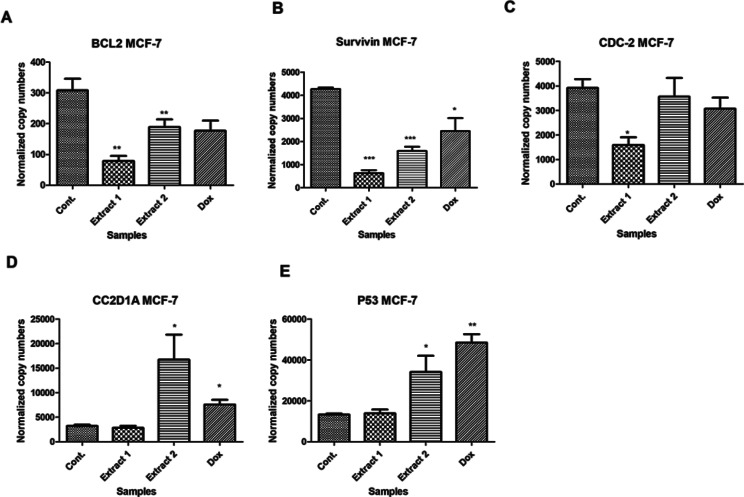
Gene expression analysis MCF-7 cells treated Doxorubicin (Dox), *Thalassia hemperchii* (extract 1) and *Enhalus ocoroides* (extract 2) for **A** (BCL 2), **B** (Survivin), **C** (CDC-2), **D** (CC2D1A) and **E** (p53). Data are expressed as mean ± SD. *** *P* < 0.001 ** *P* < 0.01 * *P* < 0.05

As illustrated in Fig. 9, the effect of Doxorubicin, *Thalassia hemperchii* (extract 1) and *Enhalus ocoroides* (extract 2) on Bcl-2, survivin, CDC2, CC2D1A, and p53 genes expression levels were evaluated by normalization of their expression to that of β-Actin and in comparison to negative control cells. According to Yahya et al. [44]. It is well known that p53 genes are down-regulated whereas Bcl-2, survivin, and CDC2 expression levels are up-regulated in breast cancer cell lines. In the current study, in MCF-7, Doxorubicin, *Thalassia hemperchii* (extract 1), *Enhalus ocoroides* (extract 2) exhibited a marked reduction in Bcl-2, Survivin and CDC2 gene expression levels but significantly increased the expression of p53 and CC2D1A as compared to control cells (Fig. 9A, B, C, D &E). These finding may introduce these extracts as a promising regulators of apoptosis and cell cycle.

On the other side, *Enhalus ocoroides* produced significant increase in p53 as compared to control cells which is considered as an important tumor suppressor and transcription factors. p53 has an important role in various cellular processes, such as cellular response to DNA damage, cell signal transduction, genomic stability, apoptosis and cell cycle arrest [45].

## Discussion

Natural remedies have been extensively used to cure a number of illnesses and are now a significant field of study for the development of new cancer drugs [46–48]. This growing attention was due to the high curative rate and as well as the minimal side effect.

Valuable medicinal plants are under pressure and their biodiversity is at risk due to the growing demand for medications generated from plants [49]. In developing nations, the endangerment of species is a result of growing populations and deforestation. Plant part replacement, tissue cultures, cryopreservation, and germplasm conservation must all be implemented in order to support the conservation of these species [50]. Conservation may also be aided by the mass production of therapeutic plant species and the industrial use of their raw byproducts [51]. Seagrass were reported as a valuable source of phenolic compounds and are of ecological importance [17]. The sustainability of any marine organism can’t be granted because their abundance is based on different environmental factors. However, seagrass can be grown in tissue culture labs. The cytotoxic effect of phenolic compounds is induced through the modulation of various mechanisms, such as cellular proliferation, differentiation, apoptosis, angiogenesis, and metastasis. These products have demonstrated anti-carcinogenic activities by obstructing the onset, development, and progression of cancer [52]. Because it is a more affordable option than traditional cancer therapy, this idea is gaining more attention. Diverse groups of phenolic acids are abundant in marine seagrasses [15]. Caffeic acid and kaempferol, in particular, are phenolic acids that have anticancer, anti-inflammatory, antibacterial, immune-regulatory, and antioxidant properties [53, 54]. Several investigations have verified their biological impact on cancerous cells. The cytotoxicity activity and the capacity to prevent migration in the colorectal cancer cell lines, SW480 and SW620, were demonstrated by Villota et al. [55]. Coffee extract containing Caffeic and Chlorogenic acid has also been observed to induce apoptosis and regulate the cell cycle in prostate cancer cells [56] and K562 chronic myeloid leukemia cells [57].

The phytochemical results show the difference in the chemical composition of *Enhalus acoroides* and *Thalassia hemprichii*. with predominance of more phenolic compounds functional groups (carboxylic acid, phenols, alcohols) in *Thalassia hemprichii* (Ehren.) Asch. explaining the difference in their biological activity against cancer cell lines. *Thalassia hemperchii* is more efficient with IC_50_ value 101.19 µg/ml. On the other hand, *Enhalus acoroides* showed proliferation inhibition on the growth of MCF-7 with IC_50_ values of 604.2 µg/ml. Apoptosis (programmed cell death) is triggered via both extrinsic and intrinsic pathways in mammalian cells. The mitochondrial (intrinsic) apoptotic pathway is mainly regulated by the Bcl‑2 family proteins (pro‑apoptotic and anti‑apoptotic proteins) such as Bax and Bcl‑2 [58]. Our data showed that both extracts TH and EA induce apoptosis and cell cycle arrest at G0/S phase. The result was aligned with the capacity of the extracts to induce disruption in the mitochondrial membrane potential suggesting that sea grasses extracts induces apoptosis via the mitochondrial apoptotic pathway. The apoptotic cascade in the treated cells was presumably set off by this decline in mitochondrial membrane potential [59, 60].

Various studies referred to the apoptotic effect of phytonutrients including flavonoids, phenolic acids and carotenoids. The apoptotic activity is mainly associated with the down-regulation of Bcl-2 expression in breast cancer cell lines [61, 62]. Additionally, the immunoblotting results shown revealed that *Thalassia hemperchii* and *Enhalus acoroides* significantly up-regulated the expression level of p53 but down-regulated the expression of Bcl2. p53 play an important role in the transition from normal cell to abnormal tumor cell and it has been known as a common mutated cancer suppressor in human tumorigenesis [63]. The increase of p53 and p21 gene expression and the reduction of CDK2 gene expression were the mechanisms of action, which may result in G0/G1 cell cycle arrest [64].

Gene expression analysis showed that p53 gene was down-regulated whereas Bcl-2, survivin, and CDC2 expression levels were up-regulated in breast cancer cell lines. Bcl-2 is a well-established anti-apoptotic protein; Bcl-2 was involved in caspase-3 initiated apoptosis. Besides, the increase in the Bax/Bcl-2 ratio promotes apoptotic events including the up-regulation of caspase 3/9 and the consequent degradation of intracellular substrates [65]. Survivin is a vital member of the inhibitor of apoptosis protein family (IAP) where it suppresses apoptosis and enhances cell division. The knockdown of survivin protein was found to suppress proliferation and enhances cell cycle arrest in Hela cells [66]. CDC2 is implicated in the progression cell cycle from the G2/M phase and consequently, anticancer agents can initiate G2/M arrest in cancer cells by inhibiting CDC2 protein levels [67]. The blocking of this step is a vital target in cancer treatment. P53 is an important player in many cellular processes, such as cellular response to DNA damage, cell signal transduction, genomic stability, apoptosis and cell cycle arrest. In addition to, its activation of p21WAF1 and Bax transcription resulting in promoting apoptosis [68]. CC2D1A, also known as Akt kinase–interacting protein 1 (Aki1) or Freud1 was reported as a scaffold protein in the PI3K–PDK1–Akt pathway. Its role in cancer biology has many controversies. It was reported that CC2D1A contributes to cell survival and proliferation through the formation of a complex with EGFR and Akt upon EGF stimulation. In Diffuse Malignant Mesothelioma, CC2D1A silencing resulted in tumor growth inhibition in mice models [69]. CDC2 protein complex is one of the kinases responsible for Aki1 phosphorylation during mitosis [70]. However, recently, Reiff et al. [71], introduced CC2D1A as tumor Suppressor gene working with the aid of Notch signaling.

## Conclusion

In conclusion, the Methanolic extract of seagrass, *Thalassia hemperchii* and *Enhalus ocoroides* collected from Wadi El Gemal National Park, Red Sea, Egypt are able to induce the intrinsic pathway of apoptosis in MCF-7 cells. Our study aims to compare between two different extracts and correlate their anticancer activity to their chemical profile. This growth inhibitory effect of the Methanolic extracts was studied by apoptosis and cell cycle assay by flowcytotmry. The data showed that extracts induced programmed cell death primarily via apoptosis as confirmed by the exteriorization of phosphatidylserine. Moreover, they have ability to induce G0/S cell cycle arrest in MCF-7. The data showed the depletion in mitochondrial membrane potential (ΔψM) in the treated cells dose-dependently. Caspases 3/7activities increased following 24 h treatment. Finally, Gene expression analysis for both of them exhibited a marked reduction in Bcl-2, Survivin and CDC2 gene expression levels but significantly increased the expression of p53 and CC2D1A as compared to control cells. This study reveals the beneficial importance of sea grasses as a source of anticancer agents. It is confirmed from our current study that these sea grasses possess active molecules such as Caffeic acid, Chlorogenic acids, catechin and kaempferol in considerable amount that might be responsible for these anticancer effects, which offering potential therapeutics and a starting point for further research using isolated active compounds against breast cancer cells as well as in *vivo* study.

### Electronic supplementary material

Below is the link to the electronic supplementary material.

Supplementary Material 1

## Data Availability

All data generated or analyzed during this study are included in this published article.

## Abbreviations

HPLC: High performance liquid chromatography.
TH: *Thalassia hemperchii*
EA: Enhalus acoroides (L.f.) Royle.
MeOH: Methanol
KBr: Potassium bromide.
MeOH: Methanol
FT-IR: Fourier transform infrared.
DPBS: Dulbecco′s Phosphate Buffered Saline.
EEAA: Egyptian Environmental Affairs Agency.
ΔψM: Mitochondrial Membrane Potential.
Bcl-2: B-cell lymphoma 2 protein family.
DOX: Doxorubicin.
DMSO: Dimethyl sulfoxide, Mr; Molecular mass
OD: Optical density.
FBS: Fetal bovine serum.
MCF-7: Human breast cancer.
ATCC: American Type Culture Collection.
DMEM: Dulbecco’s modified Eagle’s Medium.
PI: Propidium iodide.
FITC: Fluorescein isothiocyanate.
pNA: p-nitroaniline.
Aki 1: kinase–interacting protein 1.
