# Supplementary material for: In-vitro study of cytotoxic and apoptotic potential of Thalassia hemprichii (Ehren.) Asch. And Enhalus acoroides (L.f.) Royle against human breast cancer cell line (MCF-7) with correlation to their chemical profile
Source: BMC Complement Med Ther. 2024 Jun 24;24:244. doi: 10.1186/s12906-024-04512-3 (PMC11194981; doi:10.1186/s12906-024-04512-3)
Supplement: Supplementary file 1 — Supplementary Material 1 [file 12906_2024_4512_MOESM1_ESM.pdf]

Supplementary Figures:

S1

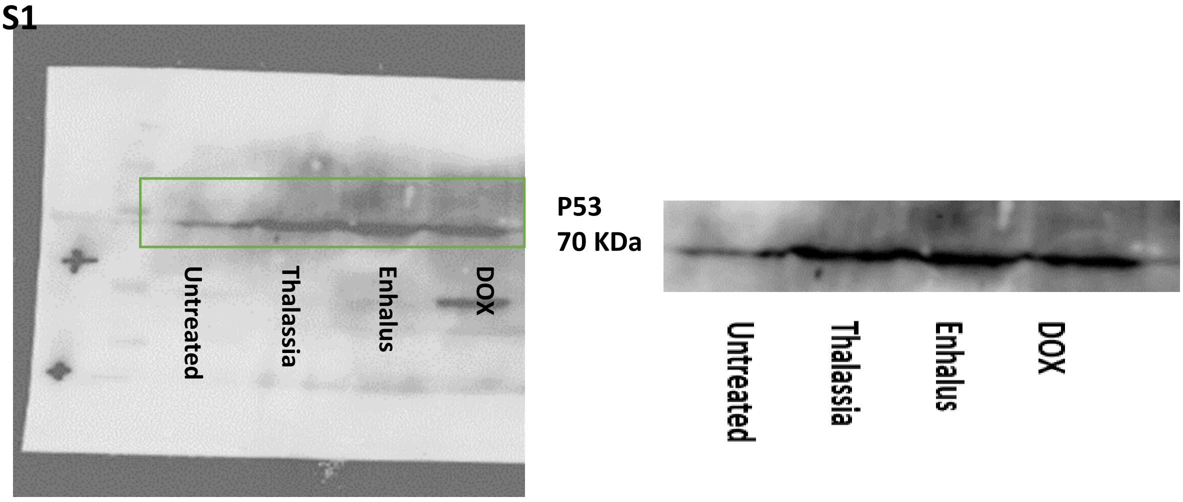

Fig S1: Raw data for protein band expression for P53 untreated, *Thalassia hemprichii* , *Enhalus acoroides* and Doxorubicin treated MCF-7

S2

BCL-2

26 KDa

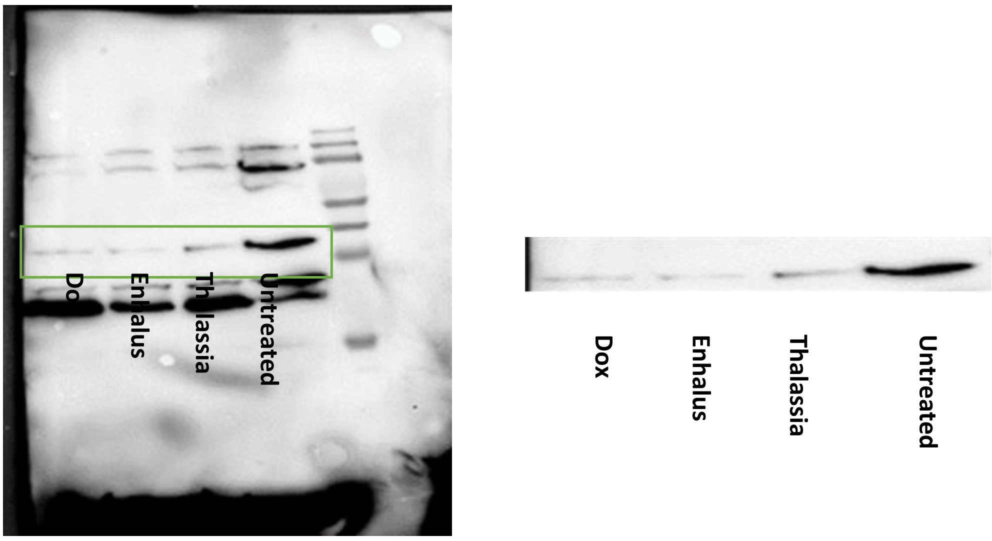

Fig S2: Raw data for protein band expression for BCL2 untreated, *Thalassia hemprichii* , *Enhalus acoroides* and Doxorubicin treated MCF-7

S3

GAPDH

36 KDa

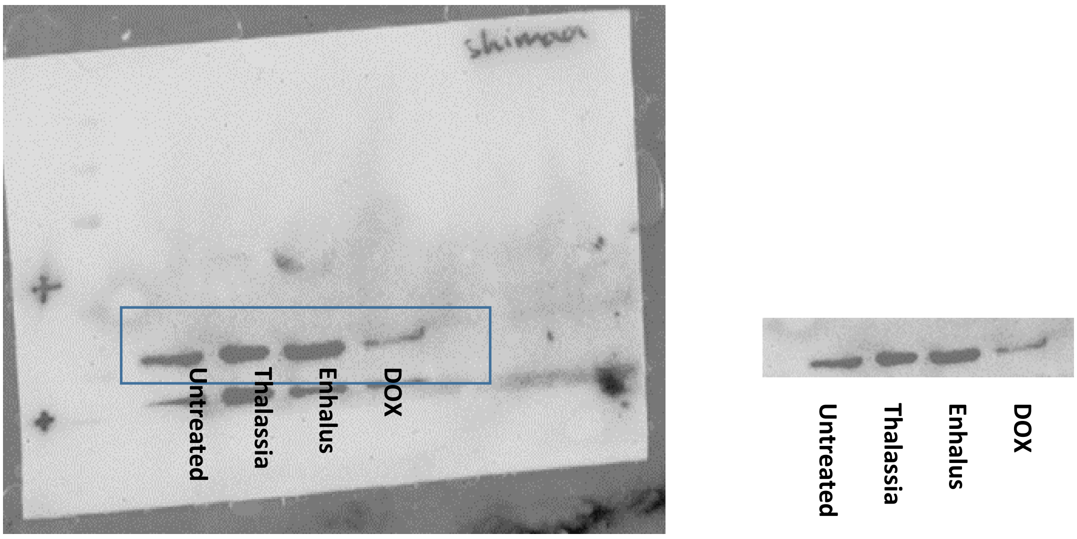

Fig S3: Raw data for protein band expression for GAPDH untreated, *Thalassia hemprichii* , *Enhalus acoroides* and Doxorubicin treated MCF-7
